# The Golden jackal (*Canis aureus*) as an indicator animal for *Trichinella britovi* in Iran

**DOI:** 10.1051/parasite/2018030

**Published:** 2018-05-10

**Authors:** Aliakbar Shamsian, Edoardo Pozio, Abdolmajid Fata, Zahra Navi, Elham Moghaddas

**Affiliations:** 1 Department of Parasitology and Mycology, Faculty of Medicine, Mashhad University of Medical Sciences, Mashhad Iran; 2 Department of Infectious Diseases, Istituto Superiore di Sanità, Rome Italy

**Keywords:** *Trichinella britovi*, Carnivore, Iran, Multiplex PCR, epidemiology

## Abstract

Nematodes of the genus *Trichinella* are zoonotic parasites causing trichinellosis. In Iran, these parasites occur in several animal species and rare cases have been recorded in humans. To monitor the epidemiological pattern of these parasites in the Khorasan-e-Razavi province, Northeastern Iran, muscle tissues were collected from the tongues of roadkill animals between 2016 and 2017: 295 stray dogs, one red fox (*Vulpes vulpes*), 12 golden jackals (*Canis aureus*), and one wild boar (*Sus scrofa*). *Trichinella* spp. larvae were retrieved using the artificial digestion method and identified to the species level by multiplex PCR. Larvae identified as *Trichinella britovi* were detected in five stray dogs (1.7%) and one golden jackal (8.3%). The results confirm the circulation of *T. britovi* in animals of the Khorasan-e-Razavi province, as previously documented. A review of the literature on *Trichinella* spp. in animals in Iran showed that these parasites were previously detected in 20.02% and 0.04% of carnivore and omnivore mammals, respectively, and that golden jackals can be screened as indicator animals for these zoonotic nematodes. Convenient sampling of *Trichinella* susceptible roadkill animals may provide a suitable method of monitoring the circulation of these parasites within any given region.

## Abstract (fr)

Les nématodes du genre *Trichinella* sont des parasites zoonotiques causant la trichinellose. En Iran, ces parasites sont présents chez plusieurs espèces animales et des rares cas humains ont été enregistrés. Pour surveiller le profil épidémiologique de ces parasites dans la province de Khorasan-Razavi, au nord-est de l’Iran, les tissus musculaires ont été prélevés sur la langue de 295 chiens errants, un renard roux (*Vulpes vulpes*), 12 chacals dorés (*Canis aureus*) et un sanglier (*Sus scrofa*), tués sur les routes, en 2016-2017. Les larves de *Trichinella* spp. ont été récupérées en utilisant la méthode de digestion artificielle et identifiées au niveau de l’espèce par PCR multiplex. Des larves identifiées comme *Trichinella britovi* ont été détectées chez cinq chiens errants (1,7%) et un chacal doré (8,3%). Les résultats confirment la circulation de *T. britovi* chez les animaux de la province de Khorasan-Razavi, comme cela a été précédemment documenté. Une revue de la littérature sur *Trichinella* spp. chez les animaux d’Iran a montré que ces parasites avaient déjà été détectés chez 20,02% et 0,04% des mammifères carnivores et omnivores, respectivement, et que les chacals dorés peuvent être criblés comme animaux indicateurs de ces nématodes zoonotiques. Un échantillonnage pratique des animaux sensibles à *Trichinella* tués sur les routes peut fournir une bonne méthode pour surveiller la circulation de ces parasites dans une région donnée.

## Introduction

Trichinellosis is a foodborne parasitic zoonosis acquired by humans through the consumption of raw or semi-raw meat infected by *Trichinella* spp. from domestic and wild swine and carnivores [11]. This disease is prevalent where these eating habits are widespread among the human population, such as those of Eastern Europe, South America, and South-East Asia [26]. Twelve taxa have been recognized within this genus so far, all of which are infectious to humans [32]. Most human infections are caused by *Trichinella spiralis* which is the most prevalent species in domestic and wild swine worldwide [26].

In Iran, human cases are rare due to religious regulations which prevent people from eating pork [18]. Pig breeding and the sale of pork by butchers are illegal in this country. However, some hunters do not respect religious regulations and slaughter wild boar and cook its meat in the same place where they hunted the animal. Sometimes, they sell wild boar meat to friends and relatives, a practice forbidden in Iran. Consequently, trichinellosis may unexpectedly emerge causing human outbreaks, like those observed in Italy [9]. In Iran in 1966, a human infection was suspected following the consumption of wild boar (*Sus scrofa*) meat [23]. More recently, a family outbreak of trichinellosis due to the consumption of wild boar meat was described near the Caspian Sea coast [17,19]. This and other outbreaks described in Algeria, Syria, and Turkey suggest that the Muslim population is at risk of acquiring trichinellosis [26].

*Trichinella* infections in wild animals have been described in Iran since 1967 [1]. These zoonotic nematodes have been detected with variable prevalence rates in carnivore mammals (brown bear, golden jackal, leopard, mongoose, stripped hyenas, jungle cat, red fox, stray dog) and omnivore mammals (Persian gerbil, wild boar) originating from six Iranian regions [1,4,13,20–22,24,25,34].

Most of the epidemiological investigations on *Trichinella* in Iranian wildlife have focused on the prevalence of *Trichinella spiralis* in different host species. In recent years, molecular epidemiological studies have shown that the species circulating in Iran was *Trichinella britovi* [4,19,21,25], previously named *Trichinella nelsoni* [28,35]. *T. spiralis* has been documented only in a golden jackal from Khuzestan in the 1980s [35].

The high prevalence of infection detected in carnivores from Gilan, Mazandaran, Isfahan, Ardabil, Khuzestan and Khorasan-e-Razavi provinces requires monitoring of *Trichinella* spp. circulation in target host species. The aims of our study were to monitor the circulation of *Trichinella* parasites in carnivore mammals in the Khorasan-e-Razavi region after a period of five years from the previous survey [4], to assess the role of canids as indicator animals for *Trichinella* spp., to identify the etiological agent at the species level, and to review the literature on *Trichinella* spp. in Iranian animals.

## Materials and Methods

### Study area and parasite collection

**Figure 1:**
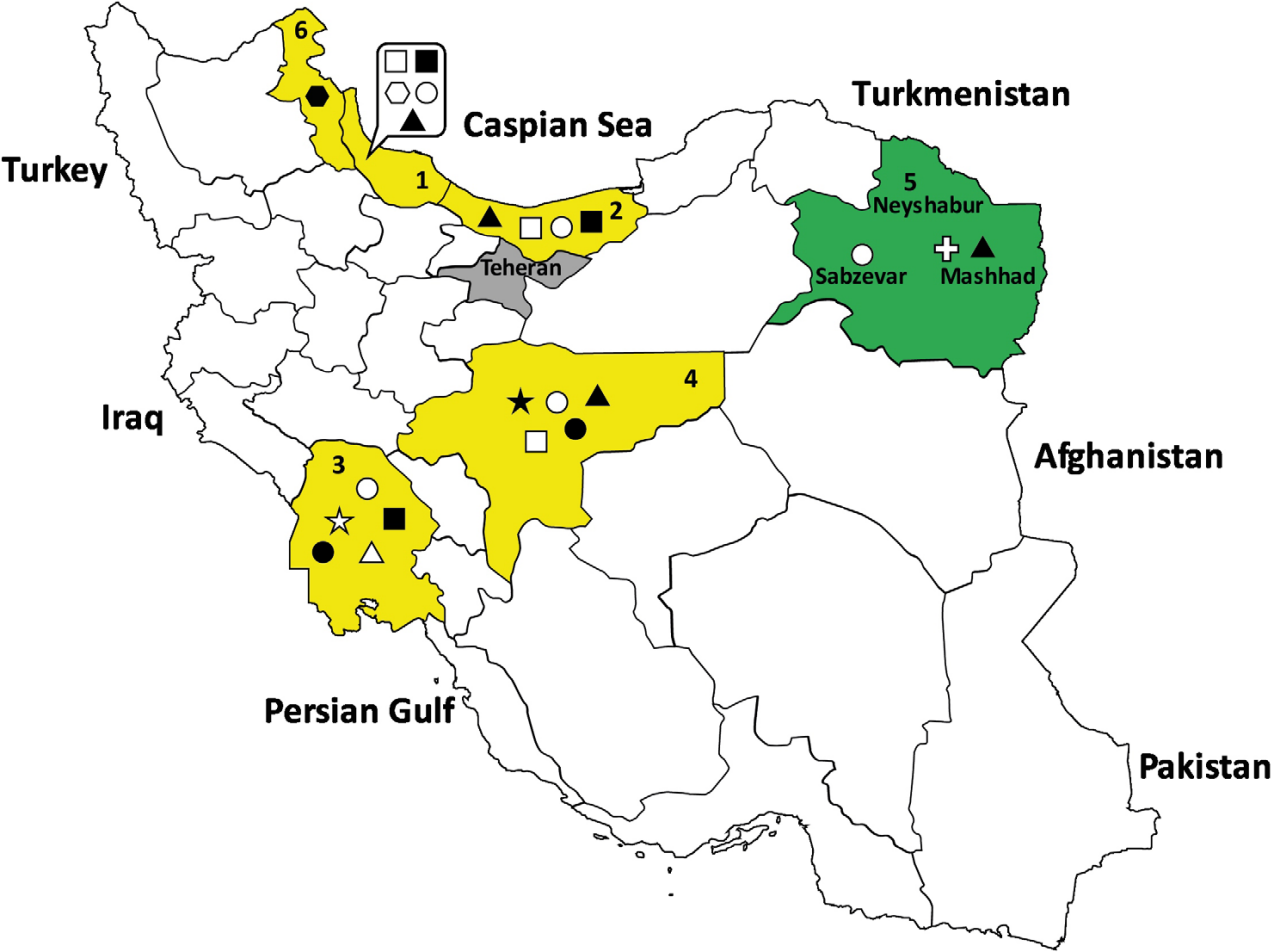
Map of Iran showing the six provinces (Gilan, 1; Mazandaran, 2; Khuzestan, 3; Isfahan, 4; Khorasan-e-Razavi, 5; and Ardabil, 6) where *Trichinella* spp. infected animals have been detected from 1967 to the present study. Khorasan-e-Razavi province, where the present study was carried out, is marked in green. In this province, animals tested for *Trichinella* spp. were collected from Mashhad, Sabzevar and Neyshabur cities. *Trichinella britovi-*positive animals were detected in the surroundings of Mashhad and Sabzevar cities. Teheran province is marked in grey. The five provinces marked in yellow show literature data. Each symbol of *Trichinella* spp.-infected animals represents from 1 to more than 100 positive heads: brown bear, open star; golden jackal, open circle; jungle cat, open hexagon; leopard, black hexagon; mongoose, open triangle; Persian gerbil, black star; red fox, open square; stripped hyena, black circle; stray dog, black triangle; wild boar, black square.

Muscle samples from the tongue were collected from 309 roadkill animals, namely 295 stray dogs, 12 golden jackals (*Canis aureus*), one red fox (*Vulpes vulpes*), and one wild boar (*Sus scrofa*) during the period 2016–2017. Sampling was carried out in the areas surrounding the cities of Mashhad (36°15’39” N, 59°36’57” E), Sabzevar (36°12’55” N, 57°40’04” E) and Neyshabur (36° 07’24” N, 58°53’06” E) in the Khorasan-e-Razavi province, Northeastern Iran (Figure 1).

Sampling and laboratory investigations were carried out under the control of the Iranian Environment Health Organization. Muscle samples collected in the field were preserved on ice during transportation to the Parasitology Laboratory, Medical Faculty of Mashhad University. Muscle samples (0.5 g) were screened by trichinoscopy at 40 x magnification. Then, positive tongues were investigated by artificial digestion of 5 g per tongue in a pool of up to 10 animals, according to a published protocol [39]. When *Trichinella* larvae were detected in the sediment, 5 g of tongue from single animals were tested separately to identify the positive animal/s. Retrieved larvae were washed and preserved in a 0.5 mL conical tube with 90% ethyl alcohol for molecular identification to the species level.

### Molecular identification of *Trichinella* larvae

*Trichinella* larvae, preserved in alcohol, were forwarded to the International *Trichinella* Reference Center (https://trichinella.iss.it/) in Rome, Italy, for species identification. Single larvae were identified using a multiplex PCR technique, according to a published protocol [31].

## Results and Discussion

*Trichinella* spp. larvae were detected in five stray dogs (1.7%) from the areas surrounding Mashhad city and in one golden jackal (8.3%) from the surroundings of Sabzevar city. The parasite load ranged from 3 to 5 larvae per gram. All the retrieved *Trichinella* larvae were molecularly confirmed as *T. britovi.*

The prevalence of *T. britovi* infections detected in the present study could be lower than the real prevalence in the investigated animals, since the initial search for *Trichinella* larvae used in this study was based on the use of trichinoscopy. It is well known that trichinoscopy has lower sensitivity than the digestion method, is strongly influenced by the analyst’s experience, and does not allow the detection of non-encapsulated larvae of the *Trichinella* genus [39].

The literature search conducted in this study revealed that most Iranian authors had identified the *Trichinella* spp. larvae isolated from wild animals as *Trichinella spiralis*, because at that time the multiple species concept of the genus *Trichinella* was unconfirmed [29]. In 1983, four *Trichinella* isolates from Iranian golden jackals were identified by crossbreeding experiments as *T. spiralis* (one isolate from Sari near the Caspian Sea) and as *T. nelsoni* (three isolates from Khuzestan province) [35]. Today, the *T. nelsoni* [5] isolates from the Palearctic region are named *T. britovi* [28–30,32]. In 2009, a *T. britovi* isolate from a wild boar, which had been the source of infection to humans, had been erroneously identified as *Trichinella murrelli* [17,19]. Excluding this discrepancy, all *Trichinella* isolates from Iranian animals identified to the species level by molecular methods were identified as *T. britovi*. However, we must consider that *Trichinella* infections have been investigated only in six out of 31 Iranian provinces; it follows that the epidemiology of *Trichinella* in about 80% of the country is still unknown.

In Iran during a 50-year period (1967-2017), *Trichinella* parasites were screened for in 1,014 carnivore and 27,157 omnivore mammals, of which 203 (20.02%) and 12 (0.04%), respectively, tested positive (Table 1). The difference in prevalence rates (20.02% versus 0.04%) of infection between carnivore and omnivore mammals is consistent with epidemiological investigations carried out in other countries where *T. britovi* is known to circulate [3,8,10,30,37]. Epidemiological data on *T. britovi* in wild animals are supported by experimental data in domestic pigs, wild boars and red foxes, which show that *T. britovi* survives longer in carnivore than in omnivore mammals [14,15,27]. *Trichinella* spp. have also been detected in other carnivore mammals (brown bear, red fox, leopard, mongoose, jungle cat and stripped hyenas) and omnivore mammals (Persian gerbil), but the number of tested animals per species (Table 1) is too low to estimate their role in the epidemiology of this parasite.

**Table 1:** *Trichinella* spp. in animals in Iran from 1973 to the present study.

Host	Province	N. positive/tested (%)	Reference
Badger (*Meles meles*)	Gilan and Mazandaran	0/1	[22]
Brown bear (*Ursus arctos*)	Khuzestan	1/16	[22]
Domestic cat	Gilan and Mazandaran	0/1	[22]
Domestic dog	Gilan and Mazandaran	0/1	[22]
Golden jackal (*Canis aureus*)	Gilan and Mazandaran	38/63 (60.31)	[22]
	Isfahan	10/18 (55.55)	[34]
	Khuzestan	11/25 (44.00)^a^	[20]
	Gilan and Mazandaran	105/125 (84.00)	[13]
	Khuzestan	1/1^b^	[35]
	Mazandaran	1/1^c^	[35]
	Khuzestan	2/18 (11.11)	[21]
	Khorasan-e-Razavi	1/12 (8.33)	present study
Insectivores	Gilan and Mazandaran	0/20 d	[22]
	Gilan and Mazandaran	0/26 e	[13]
Jungle cat (*Felis chaus*)	Gilan	2/3	[22]
	Khuzestan	0/4	[20]
Lagomorpha^f^	Gilan and Mazandaran	0/28	[13]
Leopard (*Panthera pardus saxicolor*)	Ardabil	1/1^c^	[25]
Mongoose (*Herpestes auropunctatus*)	Khuzestan	3/10	[24]
Persian gerbil (*Meriones persicus*)	Isfahan	1/29 (3.44)	[34]
Red fox (*Vulpes vulpes*)	Isfahan	2/18 (11.11)	[34]
	Khuzestan	0/6	[20]
	Gilan and Mazandaran	3/10	[13]
	Khorasan-e-Razavi	0/2	[4]
	Khorasan-e-Razavi	0/1	present study
Stripped hyena (*Hyena hyena*)	Isfahan	1/1	[34]
	Khuzestan	1/1	[20]
	Khorasan-e-Razavi	0/2	[4]
Stray dog	Teheran	0/21	[20]
	Gilan	0/1	[20]
	Gilan and Mazandaran	9/100 (9.00)	[13]
	Isfahan	2/10	[34]
	Khuzestan	0/37	[20]
	Isfahan	1/75 (1.4)	[40]
	Khorasan-e-Razavi	3/120 (2.5)	[4]
	Khuzestan	0/14	[21]
	Khorasan-e-Razavi	5/295 (1.69)	present study
Wild boar (*Sus scrofa*)	Gilan and Mazandaran	2/4,950 (0.04)	[1]
	Gilan and Mazandaran	5/21,143 (0.02)	[22]
	Khuzestan	1/4	[20]
	Gilan	1/1 g	[17]
	Khorasan-e-Razavi	0/26	[4]
	Mazandaran	2/35 (5.7)Tb	[33]
	Khorasan-e-Razavi	0/1	present study
Wild rodents	Gilan and Mazandaran	0/30^h^	[22]
	Isfahan	0/93^i^	[34]
	Gilan and Mazandaran	0/746^l^	[13]
	Khorasan-e-Razavi	0/25^m^	[4]

a larvae from two golden jackals were successively identified as *Trichinella britovi* (see [35]);

b the isolate has been identified as *Trichinella spiralis*;

c the isolate has been identified as *Trichinella britovi*;

d 20 greater white-toothed shrew (*Crocidura russula*);

e 13 hedgehogs (*Erinaceus europeus*) and 13 bicolored shrew (*Crocidura leucodon*);

f 7 hares (*Lepus capensis*) and 21 pika (*Ochotona rufescens*);

g erroneously identified as *Trichinella murrelli* (see [19]);

h house mouse (*Mus musculus*), wood mouse (*Apodemus sylvaticus*) and black rat (*Rattus rattus*);

i 43 house mouse (*Mus musculus*), 9 grey hamster (*Cricetulus migratorius*), 15 short-tailed bandicoot rat (*Nesokia indica*), 13 Sundevall’s jird (*Meriones crassus*), 2 wood mouse (*Apodemus sylvaticus*), and 11 great gerbils (*Rhombomys opimus*);

l 56 small five-toed jerboa jerboas (*Allactaga elater*), 4 dormice (*Glis glis*), 160 house mice (*Mus musculus*), 206 wood mice (*Apodemus sylvaticus*), 10 Indian scaly tailed murine rats (*Nesokia indica*), 7 rats (*Rattus ratoides*), 108 hamsters (*Cricetulus migratorius* and *Calomyscus bailwardi*), 69 voles (*Microtus transcapicus*, *M. socialis*, *M. arvalis* and M. nivalis), 130 gerbils (*Meriones persicus*, *M. crassus*, and *Rhombomys opimus*);

m species unknown.

In Iran, *T. britovi* has been documented in 2.97% of stray dogs (20/673), and in 64.25% of golden jackals (169/263) collected in five provinces (Table 1). These data suggest that the golden jackal can be a good indicator animal for the circulation of *Trichinella* spp. in Iran, due to its scavenger behavior and adaptation to different habitats, including the domestic habitat [36]. The convenient sampling of roadkill animal carcasses may represent an easy and inexpensive method of monitoring the circulation of this zoonotic parasite. The scavenger behavior of this canid allows golden jackals to be considered a sentinel for the circulation of some zoonotic pathogens, including *Trichinella* as already observed in other countries [6,7,38].

Human trichinellosis has been documented in seven individuals who had consumed wild boar meat in Iran [17,18]. However, this disease may be under-recognized due to the low numbers of ingested larvae and the lack of experience of physicians in detecting what is essentially a rare disease in Iran. In Islamic countries, butchers and hunters and their relatives can be at risk of this disease as shown by the detection of anti-*Trichinella* antibodies in 2.2% of butchers and hunters in northern Iran [16]. In Iran, hunting is a common sport from October to April. Hunters frequently grill meat immediately after hunting, a cooking method that frequently does not kill *Trichinella* larvae, especially those near bones. Even though trichinellosis is extremely rare in Islamic countries, sporadic outbreaks have been documented among certain communities belonging to other religions and cultures in Egypt, Lebanon, Israel, and Syria, or involving Muslims inadvertently like in Turkey [2,12,26]. It follows that public health, veterinary services and hunter’s associations should be aware of the circulation of these zoonotic parasites in their regions to educate hunters and consumers on the risk of acquiring this serious disease.

## Ethics

The study adhered to the tenets of the Declaration of Helsinki and was approved by the Ethics Committee at Mashhad University of Medical Sciences (Ethical code: IR.MUMS.fm.REC.1399.522).

## Funding

This work was supported by Mashhad University of Medical Sciences (Project grant 940898). The Mashhad University of Medical Sciences did not have any role in the design and execution of this investigation. The molecular identification of *Trichinella* larvae was supported by the 2017 funds of the European Commission (DG SANTE) for the European Union Reference Laboratory for Parasites, Rome, Italy.

## Authors’ contributions

AS and AF designed and coordinated the study. EM drafted and revised the manuscript. EP identified the parasite to the species level, performed the data analysis, and revised the manuscript. All authors read and approved the final manuscript.

## Conflict of Interest

There are no conflicts of interest to declare.
